# Mark4 promotes oxidative stress and inflammation via binding to PPARγ and activating NF-κB pathway in mice adipocytes

**DOI:** 10.1038/srep21382

**Published:** 2016-02-18

**Authors:** Zhenjiang Liu, Lu Gan, Yizhe Chen, Dan Luo, Zhenzhen Zhang, Weina Cao, Zhongjie Zhou, Xueting Lin, Chao Sun

**Affiliations:** 1 College of Animal Science and Technology, Northwest A&F University, Yangling, Shaanxi, 712100, China

## Abstract

MAP/Microtubule affinity-regulating kinase 4 (Mark4) plays an important role in the regulation of microtubule organization, adipogenesis and apoptosis. However, the role of Mark4 plays in oxidative stress and inflammation are poorly understood. In this study, we found *Mark4* was induced by high fat diet (HFD) while *PPARγ* was elevated significantly in mice adipocytes. Further analyses revealed Mark4 impaired mitochondrial oxidative respiration and increased reactive oxygen species (ROS) production. At same time, the activities of superoxide dismutase (SOD), catalase (CAT), glutathione peroxidase (GPx) were greatly reduced. By treating cells with H_2_O_2_ and vitamin E (VE), Mark4 accentuated oxidative stress along with increased mRNA level of *inflammatory factor interleukin-6* (*IL-6*) and decreased *leptin* mRNA. Furthermore, we found PPARγ bind to Mark4 promoter region and inhibited *Mark4* expression. We showed PPARγ interacted with Mark4 and inhibited the stimulating effect of Mark4 on oxidative stress and inflammation. Finally, we demonstrated that the IKKα/NF-κB signal pathway was involved in Mark4 induced oxidative stress and inflammation, while PTDC, a special inhibitor of NF-κB signal pathway, reduced oxidative stress and inflammation. Thus, our study indicated that Mark4 was a potential drug target for treating metabolic diseases.

Microtubules affinity regulated kinase 4 (Mark4), one of the microtubule affinity-regulating kinases (MARKs) family member, is expressed in multiple tissues^1^. These family members share similar structure, which can be divided into three parts: N the catalytic area, C side sequence and a combination of ubiquitin domain^2^. The functions of Mark2 and Mark3 are to regulate body glucose homeostasis and energy metabolism in knockout mice^3^. Furthermore, studies indicate that Mark4 is the negative regulator of mTORC1 which plays a central role in cell growth^4^,^5^. Recently, Mark4 knockout mice is resistance to high-fat diet (HFD) induced obesity and insulin resistance^6^. Feng *et al.* (2014) further determines that Mark4 promotes adipogenesis and triggers adipocytes apoptosis^7^. These data establish that Mark4 increases body glucose homeostasis and energy metabolism. However, the regulatory role of Mark4 on body oxidative stress and inflammation, especially in extreme obese condition, has yet to be elucidated.

Obesity, insulin resistance and type II diabetes are closely associated with chronic inflammation and characterized by abnormal cytokine production, increased acute-phase reactants, and activated a network of inflammatory signal pathways^8^. Dysfunction lipid metabolites, including free fatty acids (FFAs) and triglycerides (TGs), can induce cellular dysfunction through the production of reactive oxygen species (ROS) and the activation of inflammation^9^. Oxidative stress plays critical role in the generation of various diseases^10^,^11^. In obese individuals, oxidative stress impairs glucose uptake and decreases insulin sensitivity^12^. ROS generation also triggers cell apoptosis by directly activating the mitochondrial apoptotic pathway^13^. Additionally, our pervious data indicate that Mark4 promotes adipocytes apoptosis, thus we hypothesize that Mark4 increases adipose oxidative stress. Also inflammation is associated with cell apoptosis^14^. Oxidative stress activates a variety of transcription factors including NF-κB, AP-1, p53, PPARγ, and genes including various growth factors, inflammatory cytokines, chemokines, and cell cycle regulatory molecules^15^. Moreover, expression of PPARγ is an early and pivotal event in the differentiation of adipocytes^16^. Thiazolidinediones, the potent and selective agonist of PPARγ, promotes adipocyte differentiation in pre-adipocytes and mesenchymal stem cell lines^17^. PPARγ is also a transcriptional factor suppressing the production of inflammatory mediators^18^. To date, the regulatory role of Mark4 in oxidative stress and chronic inflammation is still unknown.

In this study, we found that Mark4 further increased the oxidative stress induced by H_2_O_2_. Mark4 also accentuated adipose inflammation which induced by high glucose concentration. In addition, we found PPARγ and Mark4 interacted directly to inhibit adipose oxidative stress and inflammation. These findings illustrate a novel function of Mark4 in the regulation of cell oxidative stress and energy balance, and Mark4 may serve as a potential drug target for treating metabolic syndrome.

## Results

### *Mark4* expression is increased along with adipose oxidative stress and inflammation

**Figure 1:**
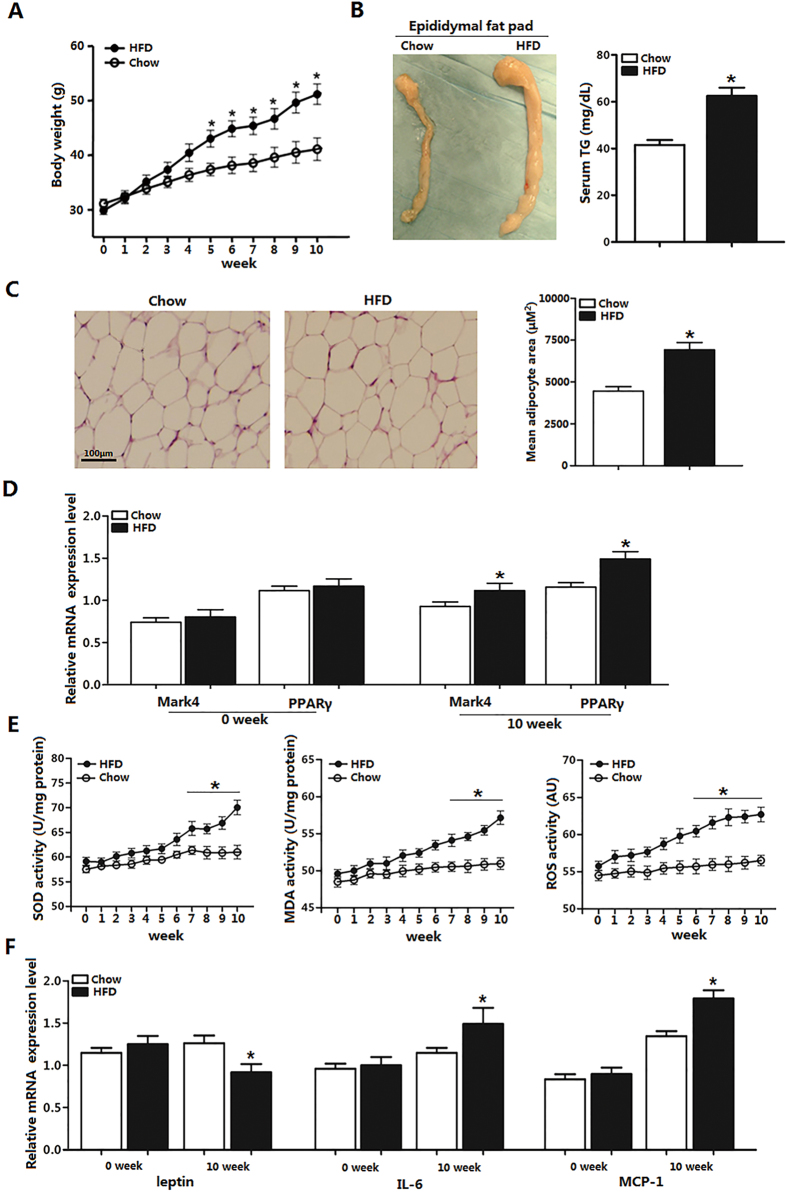
*Mark4* expression is increased along with adipose oxidative stress and inflammation. (**A**) Body weight of male mice fed HFD (n = 20 each). (**B**) EF pad representative picture of male mice fed HFD for 10 weeks. Mice serum TG content in both groups (HFD and chow diet, n = 16). (**C**) Representative hematoxylin and eosin (H&E) staining of EF pad tissue. And mean adipocyte area size (n = 16). (**D**) Relative mRNA level of *Mark4* and *PPARγ* fed chow diet and HFD on 10th week (n = 16 each). (**E**) Activity of SOD, MDA, and ROS fed on chow diet and HFD for 10 weeks (n = 16 each). (**F**) Relative mRNA level of *leptin*, *IL-6* and *MCP-1* fed on chow diet and HFD on 10th week (n = 16 each). Values are means ± SD. vs. control group, *p < 0.05.

To study the effects of high fat diet (HFD) on *Mark4* expression, we fed HFD to six-week-old male mice. Body weight was increased during 10 weeks HFD feeding, and the epididymal fat pad is 80% higher than that of chow diet fed mice (Fig. 1A,B). Tissue histology determination revealed that adipocyte size was larger in HFD mice (Fig. 1C). Moreover, serum TG level was higher in HFD group (Fig. 1B). With 10 weeks HFD feeding, we found *Mark4* mRNA level was elevated along with mRNA level of *PPARγ* (Fig. 1D). Since HFD disrupted body metabolism, we then examined the effect of HFD on oxidative stress and adipose inflammation, which showed HFD increased the activities of SOD, MDA and ROS (Fig. 1E). These changes were also associated with elevated *IL-6* mRNA and *MCP-1* mRNA, and reduced *leptin* mRNA (Fig. 1F). Thus, we concluded HFD induced energy imbalance, elevated *Mark4* and *PPARγ mRNA* levels, and led to oxidative stress and adipose inflammation.

### Mark4 blocks mitochondrial oxidative respiration in mice adipocytes

**Figure 2:**
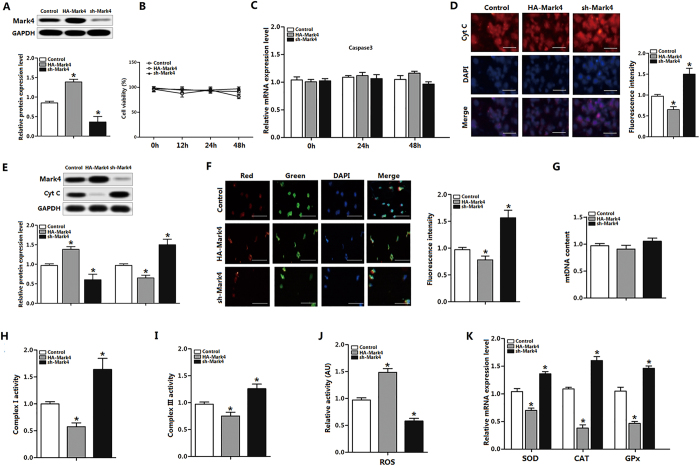
Mark4 blocks mitochondrial oxidative respiration in mice primary adipocyte. (**A**) Mark4 transfection efficiency detection in Control group, HA-Mark4 group and sh-Mark4 group after 48 h transfection (n = 3). (**B**) Cell viability measurement in Control group, HA-Mark4 group and sh-Mark4 group after transfection with HA-Mark4 and sh-Mark4 for 12 h, 24 h and 48 h (n = 3). (**C**) mRNA level of *Caspase3* after transfection with HA-Mark4 and sh-Mark4 for 48 h (n = 3). (**D**) Cyt C immunofluorescent staining after transfection with HA-Mark4 and sh-Mark4 for 48 h in primary adipocyte isolated from WAT of chow diet fed mice, and the detection of fluorescence intensity in control group, HA-Mark4 group and sh-Mark4 group. Scale bar: 100 μm (n = 3). (**E**) Immunoblots of Mark4 and Cyt C after transfection with HA-Mark4 and sh-Mark4 for 48 h in primary adipocyte (n = 3). (**F**) Immunofluorescent of JC-1 under a fluorescence microscope after transfected with HA-Mark4 and sh-Mark4 for 48 h in primary adipocyte isolated from WAT of chow diet fed mice, and the detection of fluorescence intensity in control group, HA-Mark4 group and sh-Mark4 group. Scale bar: 100 μm (n = 3). (**G**) Copy number of mtDNA after transfection with HA-Mark4 and sh-Mark4 in primary adipocyte for 48 h (n = 3). (**H**,**I**) After transfection with HA-Mark4 and sh-Mark4 for 48 h, the activity of mitochondrion complex I, III in primary adipocyte (n = 3). (**J**) The relative activity of ROS after transfection with HA-Mark4 and sh-Mark4 for 48 h in primary adipocyte (n = 3). (**K**) Relative mRNA levels of *SOD*, *CAT*, and *GPx* after transfection with HA-Mark4 and sh-Mark4 for 48 h in primary adipocyte. The level of total GAPDH was determined as loading control. Control: no transfection group, HA-Mark4 group: overexpression of Mark4 group, sh-Mark4 group: knock down of Mark4 group. Values are means ± SD. vs. control group, *p < 0.05.

We first determined the transfection efficiency of *Mark4. Mark4* protein increased in HA-Mark4 group, while sh-Mark4 treatment reduced Mark4 protein (Figs 2A and S1). Transfection of Mark4 for 48 h did not alter cell viability and *Caspase3* mRNA significantly (Fig. 2B,C). Immunofluorescence assay for Cyt C showed that Mark4 reduced Cyt C content; this result was also confirmed by western blot analysis (Fig. 2D,E). Mitochondrial membrane potential, which represented the oxidative respiration level, was decreased in HA-Mark4 group (Fig. 2F). The mtDNA was unchanged by HA-Mark4 treatment (Fig. 2G). Consistent with reduced mitochondrial oxidative respiration, the activities of mitochondrial Complex I and III were also decreased in HA-Mark4 group (Fig. 2H,I). Overexpression of Mark4 elevated ROS level and reduced mRNA of *SOD*, *CAT* and *GP*_*X*_ (Fig. 2J,K). Overlapping results were obtained in the cells transfected another two shRNAs of Mark4, thus excluding the off-target effects of shRNA treatment (Fig. S5). Thus, our data clearly showed that Mark4 increased mitochondrial oxidative respiration but had no effect on mitochondrial biogenesis.

### Mark4 promotes adipose oxidative stress

**Figure 3:**
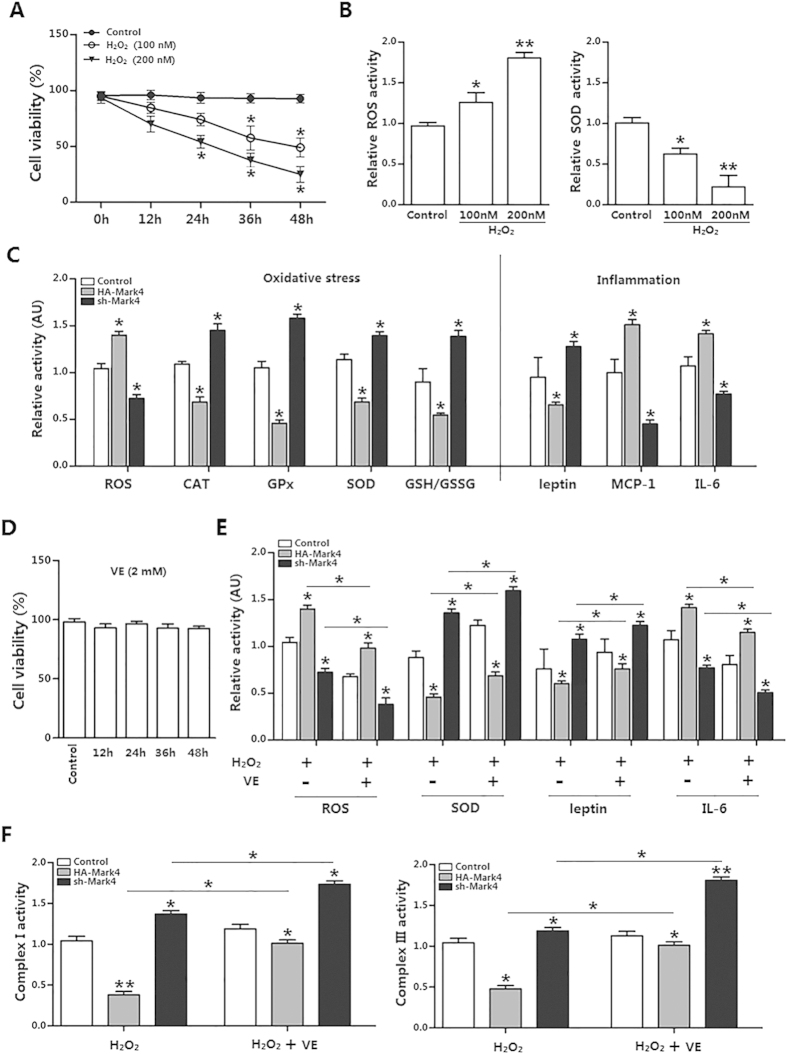
Mark4 promotes adipose oxidative stress. (**A**) Primary adipocytes isolated from WAT of chow fed diet mice were cultured and incubated for 0 h, 12 h, 24 h, 36 h and 48 h in the presence of 100 nM or 200 nM H_2_O_2_. Cell viability was detected by CCK-8 (n = 3). (**B**) The relative ROS and SOD activity of the primary adipocytes incubated for 24 h in the presence of 100 nM H_2_O_2_ (n = 3). (**C**) Relative activity of ROS, CAT, GPx, SOD, GSH/GSSG, leptin, MCP-1 and IL-6 after transfection with HA-Mark4 and sh-Mark4 for 48 h in primary adipocyte. Before transfection primary adipocytes were pretreated with 100 nM H_2_O_2_ for 24 h (n = 3 each). (**D**) Isolated primary adipocytes were cultured and incubated for 0 h, 12 h, 24 h, 36 h and 48 h in the presence of 2 mM VE. Cell viability was detected by CCK8 (n = 3). (**E**) Relative activity of ROS, SOD, leptin and IL-6 after transfection with HA-Mark4 and sh-Mark4 for 48 h in primary adipocyte. Before transfection primary adipocytes were pretreated with 2 mM VE or 100 nM H_2_O_2_ for 24 h (n = 3). (**F**) After transfection with HA-Mark4 and sh-Mark4 for 48 h, the activity of mitochondrion complex I, III in primary adipocyte (n = 3). Before transfection primary adipocytes were pretreated with 2 mM VE or 100 nM H_2_O_2_ for 24 h (n = 3). Control: no transfection group, HA-Mark4 group: overexpression of Mark4 group, sh-Mark4 group: knock down of Mark4 group. Values are means ± SD. vs. control group, *p < 0.05, **p < 0.01.

We next addressed whether Mark4 was involved in oxidative stress production. We first determined Mark4 transfection efficiency (Fig. S2). Then we used 100 nM H_2_O_2_ to establish the oxidative stress model (Fig. 3A). Under this stringent condition, elevated ROS production and reduced activities of CAT, GPx and SOD as well as the GSH/GSSH ratio clearly confirmed that Mark4 increased oxidative stress (Fig. 3B,C). These changes were associated with the elevated activities of MCP-1 and IL-6 and the reduced level of leptin (Fig. 3C). To further determine the role of Mark4 on adipose oxidative stress, vitamin E (VE) (2 mM) was used to alleviate the oxidative stress (Fig. 3D). Our data showed that elevated Mark4 prevent VE from alleviating oxidative stress; while reduced Mark4 allows VE to alleviate adipose oxidative stress (Fig. 3E). Moreover, elevated Mark4 also reduced mitochondrial Complex I and III, further establishing the role of Mark4 on oxidative stress (Fig. 3F).

### Mark4 aggravates inflammation response in mice adipocytes

**Figure 4:**
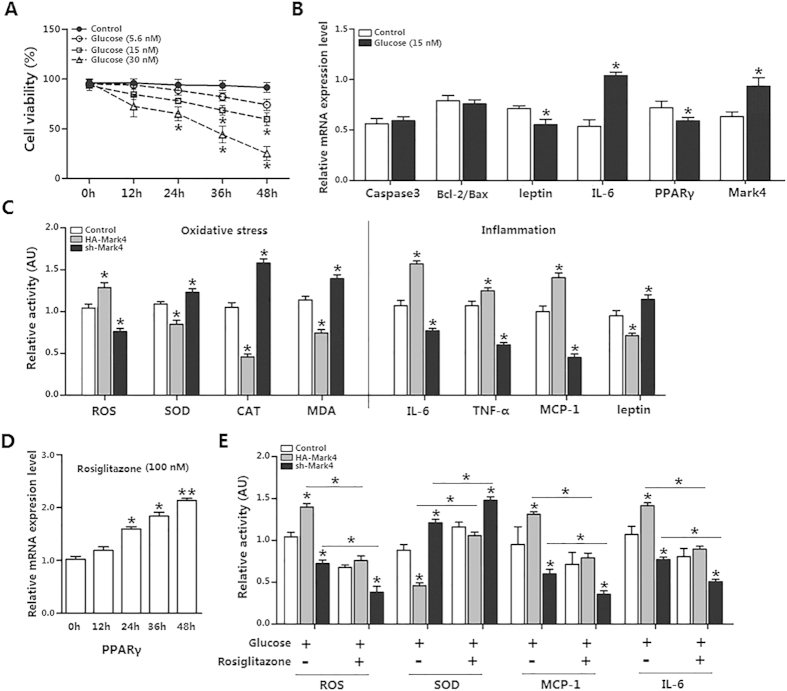
Mark4 aggravates inflammation response in mice adipocytes. (**A**) Primary adipocytes isolated from WAT of chow fed diet mice were cultured and incubated for 0 h, 12 h, 24 h, 36 h and 48 h in the presence of 5.6 nM, 15 nM and 30 nM glucose. Cell viability was detected by CCK-8 (n = 3). (**B**) Relative mRNA expression of *Caspse3*, *Bcl-2/Bax*, *leptin*, *IL-6*, *PPARγ* and *Mark4* of the primary adipocytes incubated for 24 h in the presence of 15 nM glucose (n = 3). (**C**) Relative activity of ROS, SOD, CAT, MDA, IL-6, TNF-α, MCP-1 and leptin after transfection with HA-Mark4 and sh-Mark4 for 48 h in primary adipocyte. Before transfection, primary adipocytes were treated with 15 nM glucose for 24 h (n = 3). (**D**) Primary adipocytes were cultured and incubated for 0 h, 12 h, 24 h, 36 h and 48 h in the presence of 100 nM rosiglitazone for 24 h. Relative mRNA expression of PPARγ was detected (n = 3). (**E**) Relative activity of ROS, SOD, MCP-1 and IL-6 after transfection with HA-Mark4 and sh-Mark4 for 48 h in primary adipocyte. Before transfection primary adipocytes were pretreated with 15 nM glucose or 100 nM rosiglitazone for 24 h (n = 3). Control: no transfection group, HA-Mark4 group: overexpression of Mark4 group, sh-Mark4 group: knock down of Mark4 group. Values are means ± SD. vs. control group, *p < 0.05.

Having determined that both PPAR**γ** and Mark4 were elevated after HFD feeding, we next asked whether there was a connection between Mark4 and PPARγ in adipose inflammation response. Mark4 transfection efficiency was presented in Fig. S3A. We first incubated adipocytes using glucose (15 nM) for 24 h to establish an adipose inflammation model (Fig. 4A). Inflammation key gene *IL-6* mRNA was increased, whereas *leptin* mRNA was reduced. Although inflammation had been linked to cell death, we found cell apoptosis genes *Caspase3* and *Bcl-2/Bax* did not change significantly in this adipose inflammation model (Fig. 4B). Moreover, *PPAR**γ*** mRNA level was lower while *Mark4* mRNA was elevated (Fig. 4B). Next we examined the oxidative stress in this adipose inflammatory model. Similarly, Mark4 promoted ROS production and adipose oxidative stress (Fig. 4C). ELISA measurement of IL-6, TNF-α and MCP-1 showed that elevated Mark4 enhanced adipose inflammation response (Fig. 4C). Rosiglitazone, the potent and selective agonist of PPARγ (Fig. 4D), was used to examine the regulatory role of PPAR**γ** on Mark4 functions. Figure 4E showed that rosiglitazone reduced adipose inflammation response and oxidative stress in elevated Mark4 group (Fig. 4E). Thus, our data showed that Mark4 and PPARγ played an opposing role in adipose inflammation response and oxidative stress.

### PPARγ inhibits adipose oxidative stress and inflammation by interacting directly with Mark4

**Figure 5:**
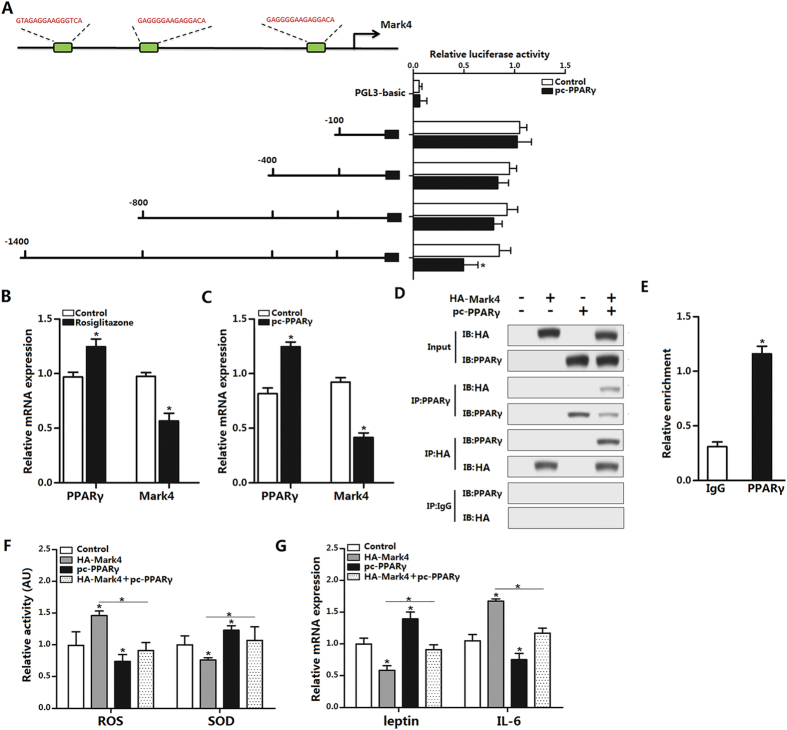
PPARγ inhibits adipose oxidative stress and inflammation by interacting directly with Mark4. (**A**) Fragments of Mark4 promoter fused to a luciferase reporter gene were co-transfected into cells together with PGL3-basic (control) or pc-PPARγ (n = 3). Luciferase activity was corrected for Renilla luciferase activity and normalized to control activity (n = 3). (**B**) Relative mRNA expression of *Mark4* and *PPARγ* of the primary adipocytes incubated for 24 h in the presence of 100 nM rosiglitazone (n = 3). (**C**) Relative mRNA expression of *Mark4* and *PPARγ* of the primary adipocytes with pc-PPARγ transfected for 48 h (n = 3). (**D**) Mark4 interacted with PPARγ. Immunoprecipitation (IP) analysis was performed in HA-Mark4 and pc-PPARγ transfected cells (n = 3). (**E**) ChIP analysis of Mark4 and PPARγ in adipocytes (n = 3). (**F**) Relative activity of ROS and SOD after transfection with HA-Mark4 and pc-PPARγ for 48 h in primary adipocyte (n = 3). (**G**) Relative mRNA of *leptin* and *IL-6* after transfection with HA-Mark4 and pc-PPARγ for 48 h in primary adipocyte (n = 3). Control: transfection of pcDNA3.1-vector group, HA-Mark4 group: overexpression of Mark4 group, pc-PPARγ: overexpression of PPARγ group. Values are means ± SD. vs. control group, *p < 0.05.

We next explored whether Mark4 and PPARγ interacted physically. With Genomatix software analysis, we found three potential binding sites of PPARγ on Mark4 promoter region (Fig. 5A). Then using luciferase reporter assay, we identified the −1400 ~ −800 region was the binding site for PPARγ (Fig. 5A). Moreover, rosiglitazone also reduced *Mark4* mRNA (Fig. 5B). To verify the inhibiting effect of PPARγ on *Mark4*, we used pc-PPARγ to treat cells, Fig. 5C indicated *Mark4* was also down-regulated (Fig. 5C). Immunoprecipitation (IP) assay showed Mark4 strongly interacted with PPARγ (Fig. 5D). The interaction between Mark4 and PPARγ was also verified by the ChIP measurement (Fig. 5E). Finally, through co-transfection of HA-Mark4 and pc-PPARγ into adipocytes, we found that Mark4 and PPARγ played opposing role on adipose oxidative stress and inflammation (Fig. 5F,G).

### IKKα/NF-κB signal is essential for Mark4 activated adipose oxidative stress and inflammation

**Figure 6:**
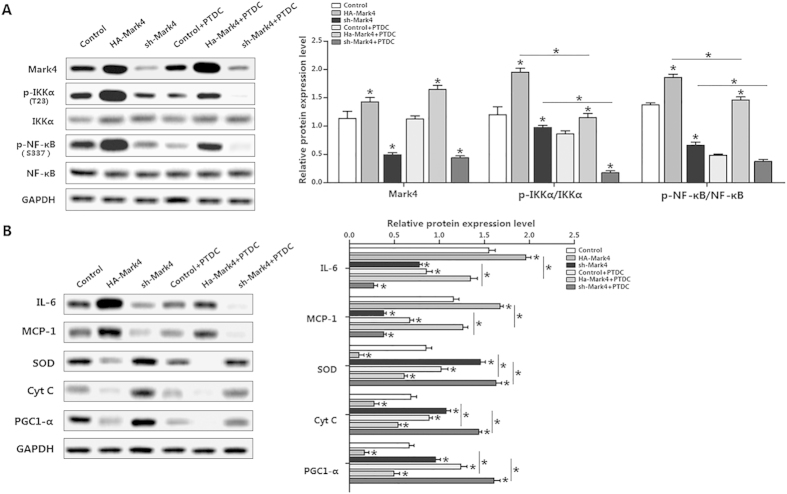
IKKα/NF-κB signal is essential for Mark4 activated adipose oxidative stress and inflammation. (**A**) Representative immunoblots and densitometric quantification for Mark4, p-IKKα (T23) and p-NF-κB (S337) after transfection with HA-Mark4, sh-Mark4 and PTDC for 48 h in primary adipocyte (n = 3). (**B**) Representative immunoblots and densitometric quantification for IL-6, MCP-1, SOD, Cyt C and PGC1-α after transfection with HA-Mark4, sh-Mark4 and PTDC for 48 h in primary adipocyte (n = 3). The level of total GAPDH was determined as loading control. Control: no transfection group, HA-Mark4 group: overexpression of Mark4 group, sh-Mark4 group: knock down of Mark4 group. Values are means ± SD. vs. control group, *p < 0.05.

We further confirmed that the IKKα/NF-κB pathway was involved in Mark4 activated adipose oxidative stress and inflammation. Specifically, overexpression of Mark4 increased the ratio of phosphorylated IKKα (T23) to total IKKα protein and accompanying elevated NF-κB phosphorylation (Fig. 6A). Similarly results were obtained in the cells overexpression another two shRNAs of Mark4, thus excluding the off-target effects of shRNA treatment (Fig. S5). Despite with PTDC treatment which is a specific NF-κB pathway inhibitor, Mark4 still increased the activity of phosphorylated IKKα (T23) (Fig. 6A). In addition, IL-6 and MCP-1 protein were both increased, along with reduced SOD, Cyt C and PGC1-α (Fig. 6B). Conversely, suppression of NF-κB by the NF-κB specific inhibitor PTDC reduced IL-6 and MCP-1 protein expression in Mark4 overexpression group (Fig. 6B). Protein expression of SOD, PGC1-α and Cyt C were also up-regulated in HA-Mark4 plus PTDC group (Fig. 6B). Mark4 dead mutant (Mark4 DA) mRNA can be translated but inactive as specific amino acids were mutated. We conducted a parallel experiment using the Mark4 dead mutant to confirm the effects of elevated Mark4 in adipose oxidative stress and inflammation. As shown in Figure S4, Mark4 DA did not change protein levels of IL-6, MCP-1, SOD and PGC1-α compared with those in HA-Mark4 group or sh-Mark4 group (Fig. S4). Collectively, our data indicated IKKα/NF-κB pathway was participated in Mark4 activated adipose oxidative stress and inflammation.

## Discussion

Mark4, the fourth paralog in the MARK/PAR-1 kinase family, is involved in the regulation of dynamic biological functions^19^,^20^,^21^. Generally, Mark4 is associated with tissue development and is required for the initiation of axoneme extension after the docking of ciliary vesicles to the mother centriole^22^. Mark4 plays a critical role in pathogenesis of many diseases such as Alzheimer’s disease and brain tumors^23^,^24^. Recently, the hypothesis that excessive lipid accumulation directly contributes to metabolic syndrome in adipose tissues has gained much attention. Our previous studies showed that Mark4 knockout mice were protected from obesity and insulin resistance (IR) induced by HFD^6^. We found that Mark4 expression and the activity of ROS were significantly elevated in HFD group, along with increased expression of inflammatory cytokines. Excessive lipid accumulation induces inflammation, mitochondrial dysfunction, and pathogenesis of IR in insulin-responsive tissues in obese rodent models and humans^25^,^26^,^27^. Our results showed that Mark4 impaired mitochondrial oxidative respiration by inhibiting Cyt C expression, reducing membrane potential and blocking the respiratory chain. In addition, ROS activity was also elevated by Mark4 overexpression. Conversely, Mark4 overexpression significantly reduced activities of SOD, CAT, GPx and MDA which are associated with anti-oxidative stress in adipocytes.

Oxidative stress plays an important role in the pathogenesis of various diseases^28^,^29^. Excessive lipid deposition increases oxidative stress causing dysregulation of adipocytokines, and consequently developing into metabolic syndrome^30^,^31^. In this study, we focus on the influence of Mark4 on mitochondrial oxidative stress. By treating adipocytes with H_2_O_2_, we induced adipose oxidative stress, as expected Mark4 aggravated this process by increasing ROS activity and reducing the activities of CAT, GPx, SOD and GSH/GSSG ratio. Although this effect was attenuated by VE, an antioxidant, Mark4 still significantly impaired mitochondrial oxidative respiration and induced oxidative stress. Many diseases including inflammation, sepsis and septic shock are associated with alterations in the production of reactive nitrogen and ROS^32^,^33^. Inflammation is a series of cellular and molecular responses that defend the body from infections or other impairments^34^,^35^. Obesity usually is associated with mild chronic inflammation and this is consistent with our data in HFD group^36^. Our results revealed that activation of oxidative stress caused by Mark4 led to increased inflammatory cytokines MCP-1, IL-6 and TNF-α secretion. Conversely, leptin expression was decreased in respond to HFD or Mark4 overexpression. Studies report that overexpression of Mark4 triggers kinds of cell reduction of cell viability^37^,^38^,^39^. We found that elevated Mark4 caused mild cell viability reduction and did not alert the mRNA of *Caspase 3* significantly in mice adipocytes. Our study indicated Mark4 remarkably accentuated adipose oxidative stress and aggravated adipose inflammation response. We speculated that the accumulation of ROS and inflammation responses will trigger adipocyte apoptosis in a further step^40^,^41^,^42^,^43^,^44^,^45^,^46^.

PPARγ is a member of the nuclear receptor family. It is a transcription factor among a diverse group of proteins which mediate ligand-dependent transcriptional activation and repression^18^,^47^. PPARγ is also highly expressed in the adipocytes and plays an anti-inflammatory role through inhibition of IKK pathway^8^,^48^. It seems that HFD increased expression of Mark4 and PPARγ independently. We also found that rosiglitazone, a PPARγ agonist, reversed Mark4 induced oxidative stress and inflammation. With bioinformatics software, PPARγ was identified as a transcription factor of Mark4, and as expected PPARγ significantly inhibited Mark4 promoter activity. By ChIP and immunoprecipitation assays, we also found that PPARγ interacted with Mark4 and attenuated Mark4 induced oxidative stress and inflammation.

The IκB kinase (IKK)/NF-κB signaling pathway plays an important role in immune regulatory functions^36^,^49^,^50^. NF-κB exists in the cytoplasm in an inactive form associated with regulatory proteins called IκB, while the phosphorylation of IκB molecules by IKK promotes their degradation. Then NF-κB translocates to nucleus and promotes target genes transcription^51^,^52^. Our data showed that Mark4 increased the phosphorylation of IKKα/NF-κB signal pathway. PTDC, a specific inhibitor of NF-κB signal pathway attenuated this effect. Mitochondrial oxidative stress and inflammation related factors were also reduced with Mark4 or PTDC treatment. This indicates IKKα/NF-κB signal pathway is involved in Mark4 regulation of adipocytes oxidative stress and inflammation. Moreover, parallel experiments were performed using Mark4-mutant, demonstrated that elevated oxidative stress and inflammation were due to Mark4 kinase activity.

**Figure 7:**
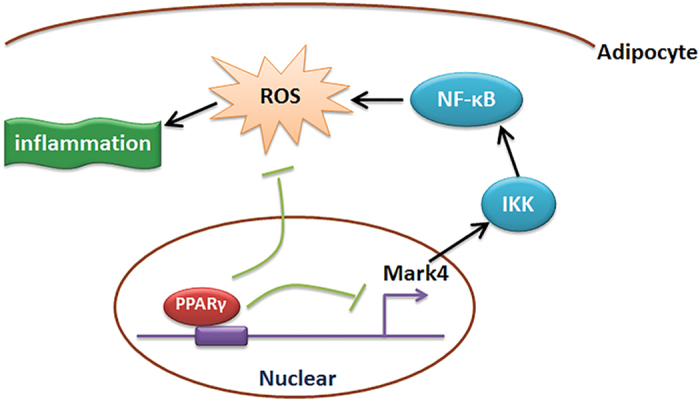
Summary of Mark4 in the regulation of oxidative stress and inflammation via IKKα/NF-κB signaling pathway in murine adipocytes. And PPARγ binding to Mark4 as a transcriptional suppressor in this regulation progress.

In conclusion, our results demonstrate that Mark4 promotes mitochondrial oxidative stress and adipose inflammation via activating IKKα/NF-κB signal pathway. Moreover, we find that PPARγ is a novel transcriptional suppressor of Mark4 and it alleviates oxidative stress and adipose inflammation by binding to Mark4 promoter region (Fig. 7). These finding shed new light on the study of molecular mechanism of metabolic disease.

## Materials and Methods

### Animal experiment

Six-week-old Kunming male mice were purchased from the Laboratory Animal Center of the Fourth Military Medical University. All mice were carried out in accordance with applicable guidelines and regulations approved by the Animal Ethics Committee of Northwest A&F University. Mice were allowed *ad libitum* access to water and standard chow laboratory diet for the first two weeks to allow them to adjust to the new environment. Mice were subsequently randomly assigned into two groups: a high-fat diet fed group (87.5% chow diet +10% lard +2% cholesterol +0.5% bile salt; Animal Center of the Fourth Military Medical University) or a chow diet fed group (Animal Center of the Fourth Military Medical University) for the next 10 weeks. Animal room was maintained under controlled conditions of temperature at 25 °C ± 1 °C, humidity at 55 ± 5%, and a 12 h light/12-dark cycle.

Body weight was recorded once a week. Serum triglyceride (TG) level was measured using the Infinity Triglyceride kit (Sigma, St. Louis, USA). H&E staining of white adipocyte was from epididymal fat pad.

### Cell culture

Epididymal white adipose tissues were harvested, visible fibers and blood vessels were removed and the adipose tissue was washed three times with PBS buffer containing 200 U/mL penicillin (Sigma, St. Louis, USA) and 200 U/mL streptomycin (Sigma, St. Louis, USA). Then the adipose tissue was minced into fine sections (1 mm^3^) with scissors and incubated in 10 mL digestion buffer containing dulbecco’s modified eagle medium (DMEM)/F-12 (Gibco, California, USA), 100 mM HEPES (Sigma, St. Louis, USA), 1.5% bovine serum albumin (Sigma, St. Louis, USA), 2 mg/mL type I collagenase (Sigma, St. Louis, USA) for 50 min at 37 °C in a water bath. After the incubation, growth medium (DMEM/F-12 (50:50)), 10% fetal bovine serum (Sigma, St. Louis, USA), 100 U/mL penicillin and 100 U/mL streptomycin were added to the digestion flask. Flask contents were mixed and filtered through nylon screens with 250 μm and 20 μm mesh openings to remove undigested tissues and large cell aggregates. The filtered cells were centrifuged at 1, 300 × g for 7 min at room temperature to separate floating adipocytes from cell pellets. Isolated cell pellets were suspended in growth medium. Finally, cells were seeded in culture plates at a density of 5 × 10^4 ^cells/cm^2^ and incubated at 37 °C under a humidified atmosphere of 5% CO_2_ and 95% air until confluence. The medium was changed every other day.

### Transfection of adipocytes with plasmids

Mark4 forced expression plasmid vector HA-Mark4 was kept in our lab. shRNA sequence against Mark4 was contrived and synthesized by Genepharma Company (Shanghai, China) using pGPU6/Neo shRNA expression vector named sh1-Mark4, sh2-Mark4 and sh3-Mark4. Then by transfection efficiency detection, the optimal shRNA of Mark4 was chosen and named sh-Mark4. Plasmids vectors used as control vectors were pcDNA3.1-vector and negative-shRNA. To exclude off-target effects of shRNA treatment, we used the other two Mark4 shRNAs, sh1-Mark4 and sh3-Mark4 which targeting different sequences of Mark4 mRNA compared with shRNA-Mark4^5^. Mark4 DA (dead mutant) was made as described previously^7^. In HA-Mark4 DA group, mark4 protein was translated inactively with specific amino acid mutations called dead mutant. 2 μg interference or expression plasmids DNA were mixed with 2 μl X-treme GENE HP Reagent (Roche, Switzerland) and Opti-MEMI media (Invitrogen, California, USA) and then added into the culture dish for 24 h or 48 h according to the protocol.

### Cell viability assay and drug treatment

Cell viability was measured by cell counting kit (CCK-8, Vazyme, China) assay. The transfected cells were seeded in 96-well plate at a density of 5 × 10^3^ and cultured for 12 h. 10 μl CCK-8 solution was added into each well and incubated for 1 hour at 37 °C. Absorbance was quantified at 450 nm by Vector 5 (Bio-TechInstruments, USA).

H_2_O_2_, Vitamin E (VE) and glucose were purchased from Sigma (St. Louis, MO, USA). H_2_O_2_ working solution (100 nM) and glucose working solution (5.6 nM, 15 nM, 20 nM) and Vitamin E (2 nM) were prepared to treat cells for 24 h before plasmids transfection.

### Measurement of Oxidative Stress

The intracellular level of ROS test was performed using a cell-permeable non fluorescent probe 2’, 7’-dichlorofluorescin diacetate (DCFH-DA) (Beyotime, Nanjing, China). The dye loading was performed by incubating the adipocytes with 10 μM DCFH-DA at 37 °C for 60 min. The production of ROS was examined using a spectrophotometer by measuring the fluorescence intensity of DCF at an excitation wavelength of 488 nm and emission wavelength of 525 nm.

For the key oxidative enzyme activity detection, cells were harvested after the medium removed, and washed with ice-cold PBS three times and lysed with cell lysis buffer. The lysate was centrifuged at 10,000 g for 5 min. Then malondialdehyde (MDA) concentration, superoxide dismutase (SOD) activity, catalase (CAT) activity, glutathione peroxidase (GP_X_) activity measurements were performed using the commercially available kits from Beyotime Co. (China). For GSH/GSSG ratio measurement, the GSH and GSSG Assay Kit (Beyotime Co. (Nanjing, China)) was used.

### Mitochondrial analysis

Fluorescent probe JC-1 (Beyotime, Nanjing, China) was used to estimate ΔΨm. Cells were incubated at 37 °C for 10 min with 5 μg/mL JC-1, then washed twice with PBS and placed in fresh medium without serum. Images were scanned by a Fluorescence Microscope (Nikon TE2000-U, Japan). The ratio of red/green fluorescent intensity was calculated.

Cyt C immunofluorescence analysis was performed after cells were washed three times with PBS, and fixed with 10% neutral formalin for 30 min and washed with PBS, then incubated with the rabbit against rat Cyt C antibody (ab133504, Abcam, Cambridge, UK) for 12 h at 4 °C. After incubation, cells were washed twice with PBS for 3 min, and then incubated for 1 h at room temperature with fluorescein isothiocyanate-conjugated goat against rabbit IgG antibody (Boster, China) diluted 1:100 in PBS, and then washed again in PBS. Finally the cells were then illuminated with the appropriate laser line and photographed with a TE2000 Nikon fluorescence microscopy (Tokyo, Japan).

mtDNA copy number was detected using QPCR method. A pair of primers for the Cox2 mtDNA region: F: TGA CAG TCC ACC TAC TTA CAA T; R: CTC CAC CAA TGA CCT GAT AT. For the adipocyte mitochondria isolation, cells were harvested and washed with cool-PBS twice, and then suspended in the ice-cold isolation buffer for 15 min. After the cells were homogenized, the homogenate was centrifuged at 1,000 g for 10 min at 4 °C, collected the supernatant and centrifuged at 11,000 g for 10 min at 4 °C. The mitochondria were collected in the sediments. The activities of the mitochondrial complexes were determined using the Mito Complex I and III Activity Assay Kits (GenMed Scientifics Inc., China).

### Enzyme-linked immunosorbent (ELISA) assay

For the IL-6, TNF-α and MCP-1 proteins detection, we used the commercial ELISA kits (R&D Systems, USA). Cells were harvested and disrupted by ultrasonication (28 KHz, 30 min).

### Immuoprecipitation (IP) and Chromatin Immunoprecipitation (ChIP) assays

Cells were transfected with HA-Mark4 plasmid, pc-PPARγ plasmid, or both. Cell lysates were obtained in RIPA buffer 48 h after transfection. Mark4 protein and PPARγ protein were immunoprecipitated using the anti-HA antibody (ab9110) and anti-PPARγ antibody separately (Abcam, Cambridge, UK). Mark4 and PPARγ were western blotted using anti-PPAR and anti-HA antibody.

Adipocytes were prepared for chromatin immunoprecipitation (ChIP) assay using a ChIP assay kit (Abcam, England) according to the manufacturer’s protocol. Primary antibodies of PPARγ (ab41928, Abcam, Cambridge, UK) or IgG (ab171870, Abcam, Cambridge, UK) were used. DNA-protein crosslinking complexes were collected, and purified DNA was subjected to qPCR with SYBR green fluorescent dye (Invitrogen, Californian, USA).

### Luciferase report assay

The mouse Mark4 promoter was amplified and inserted into the Kpn I and Hind III sites of pGL3-basic (Takara, Dalian, China). Cells were cultured in 24-well plates and co-transfected with PPARγ plasmid, HA-Mark4 plasmid or pGL3-basic plasmid (control reporter). pc-PPARγ plasmid was kept in our lab. After 48 h, cells were harvested and measured using the Dual-Luciferase Reporter assay system (Promega, USA), and luciferase activity was divided by all luciferase assay experiments, which were performed three times at least, and each conducted in triplicate.

### Real-time quantitative PCR analysis

Total RNA was extracted with TRIpure Reagent kit (Takara, Dalian, China) and 400 ng of total RNA was reverse transcribed using the M-MLV reverse transcriptase kit (Takara, Dalian, China). Primers for *Mark4*, *PPARγ*, *leptin*, *IL-6*, *MCP-1*, *SOD*, *CAT*, *GP*_*X*_, *Caspase 3*, *Bcl-2* and *Bax* were synthesized by Shanghai Sangon Ltd (Shanghai, China). Quantitative PCR was performed in 25 μL reactions containing specific primers and SYBR Premix EX Taq (Takara, Dalian, China). The levels of mRNAs were normalized to β-actin. The expression of genes was analyzed by method of 2^−△△Ct^.

### Protein extraction and Western blot Analysis

Cells were lysed in RIPA buffer for 40 min at 4 °C. Removing insoluble material by centrifugation at 12,000 × g for 15 min at 4 °C, and the supernatants were used to assay protein levels. Protein samples (50 μg) were separated by electrophoresis on 12% and 5% SDS-PAGE gels using slab gel apparatus and then transferred to PVDF nitrocellulose membranes (Millipore, USA) blocked with 5% skim milk powder/Tween 20/TBST at room temperature for 2 h. Primary antibodies against Mark4 (ab124228) and GAPDH (ab181602) were purchased from Abcam (Cambridge, UK). Antibodies against IL-6 (ab6672), PGC1-α (ab54481), SOD (ab8866), MCP-1 (ab25124), IKKα (ab32041), phospho-IKKα (T23) (ab38515), NF-kB (ab32360), phospho-NF-kB (S337) (ab28849) and Cyt C (ab133504) were all form Abcam (Cambridge, UK). And NF-κB specific inhibitor PTDC was from Sigma (St. Louis, USA). Membranes were incubated with primary antibodies at 4 °C overnight and then incubated with the appropriate HRP-conjugated secondary antibodies (Boaoshen, China) for 2 h at room temperature. Proteins were visualized using chemiluminescent peroxidase substrate (Millipore, USA), and then the blots were quantified using ChemiDoc XRS system (Bio-Rad, USA) and Quantitative analysis of immune-blotted bands was performed using Quantity One software (Bio-Rad, USA).

### Statistical analysis

Statistical analyses were conducted using SAS v8.0 (SAS Institute, Cary, NC). Data were analyzed using one-way ANOVA. Comparisons among individual means were made by Fisher’s least significant difference (LSD). Data were presented as mean ± SD. *p* < 0.05 was considered to be statistically significant.

## Additional Information

**How to cite this article**: Liu, Z. *et al.* Mark4 promotes oxidative stress and inflammation via binding to PPARγ and activating NF-κB pathway in mice adipocytes. *Sci. Rep.* **6**, 21382; doi: 10.1038/srep21382 (2016).

## Supplementary Material

Supplementary Information
