# Supplementary material for: Mark4 promotes oxidative stress and inflammation via binding to PPARγ and activating NF-κB pathway in mice adipocytes
Source: Sci Rep. 2016 Feb 18;6:21382. doi: 10.1038/srep21382 (PMC4766853; doi:10.1038/srep21382)
Supplement: Supplementary Information [file srep21382-s1.doc]

**Supplement Information**

**Mark4 promotes** **oxidative stress and inflammation via binding to PPARγ and activating NF-κB pathway** **in mice adipocytes**

Zhenjiang Liu, Lu Gan, Yizhe Chen, Dan Luo, Zhenzhen Zhang, Weina Cao, Zhongjie Zhou, Xueting Lin, Chao Sun

**Supplement Experiment**

**Figure S1:**

**
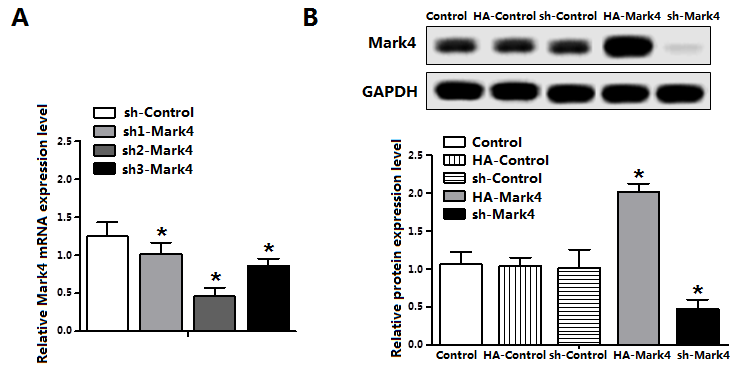
**

**Fig.S1 The selection of sh-Mark4 vector and Mark4 transfection efficiency detection (Related to Fig.2).** (A) Relative mRNA expression level of Mark4. Small-interfering RNA against Mark4 was designed based on the principle of shRNA construction and was synthesized by Invitrogen Company (Shanghai, China) using pGPU6/Neo shRNA expression vector (Genepharma, China). To exclude off-target effects of shRNA treatment, three shRNAs of Mark4 was constructed and transfected into adipocytes for 24h (n=3). (B) Relative Mark4 protein expression level. Adipocytes were transfected with HA-Control, sh-Control, HA-Mark4 and sh-Mark4 plasmids for 48h (n=3). Control group: no transfection group, HA-Control: transfected with pcDNA3.1-vector group, sh-Control: transfected with negative-shRNA group, HA-Mark4: transfected with pcDNA3.1-HA-Mark4 group, sh-Mark4: transfected with Mark4 interference plasmid group. The level of total GAPDH was determined as loading control. Values are means ± SD. vs. control group, * *p* < 0.05.

**Figure S2:**

**
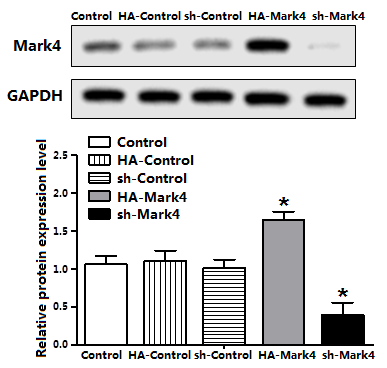
**

**Fig.S2 The Mark4 transfection efficiency detection (Related to Fig.3).** (A) Relative Mark4 protein expression level. Adipocytes were transfected with HA-Control, sh-Control, HA-Mark4 and sh-Mark4 plasmids for 48h (n=3). Control group: no transfection group, HA-Control: transfected with pcDNA3.1-vector group, sh-Control: transfected with negative-shRNA group, HA-Mark4: transfected with pcDNA3.1-HA-Mark4 group, sh-Mark4: transfected with Mark4 interference plasmid group. Values are means ± SD. vs. control group, * *p* < 0.05.

**Figure S3:**

**
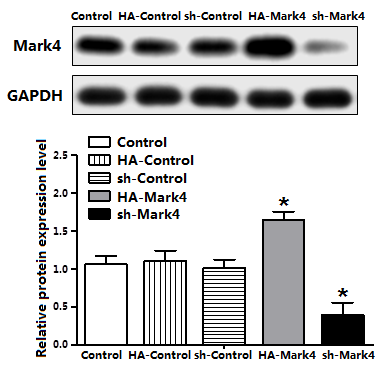
**

**Fig.S3 The Mark4 transfection efficiency detection (Related to Fig.4).** (A) Relative Mark4 protein expression level. Adipocytes were transfected with HA-Control, sh-Control, HA-Mark4 and sh-Mark4 plasmids for 48h (n=3). Control group: no transfection group, HA-Control: transfected with pcDNA3.1-vector group, sh-Control: transfected with negative-shRNA group, HA-Mark4: transfected with pcDNA3.1-HA-Mark4 group, sh-Mark4: transfected with Mark4 interference plasmid group. Values are means ± SD. vs. control group, * *p* < 0.05.


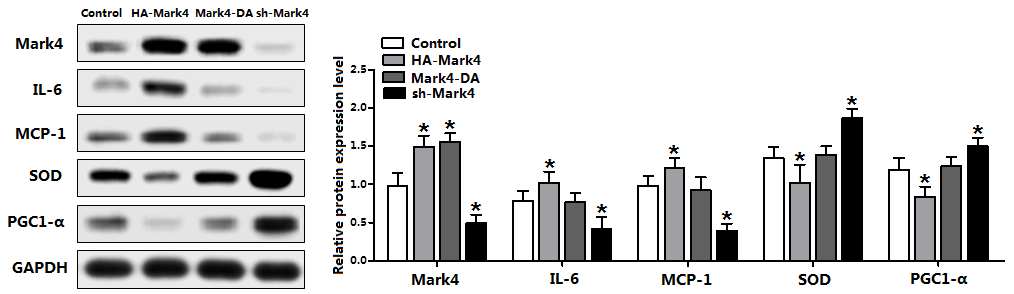
**Figure S4:**

**Fig.S4 Relative protein expression levels in adipocytes transfected with HA-Mark4, Mark4-DA and sh-Mark4 for 48h (Related with Fig.6).** The protein levels of Mark4, IL-6, MCP-1, SOD and PGC1-α were measured (n=3). Control group: no transfection group, HA-Mark4: transfected with pcDNA3.1-HA-Mark4 group, sh-Mark4: transfected with Mark4 interference plasmid group, Mark-DA: transfected with Mark4 dead mutant. Values are means ± SD. vs. control group, * *p* < 0.05.

**Figure S5:**


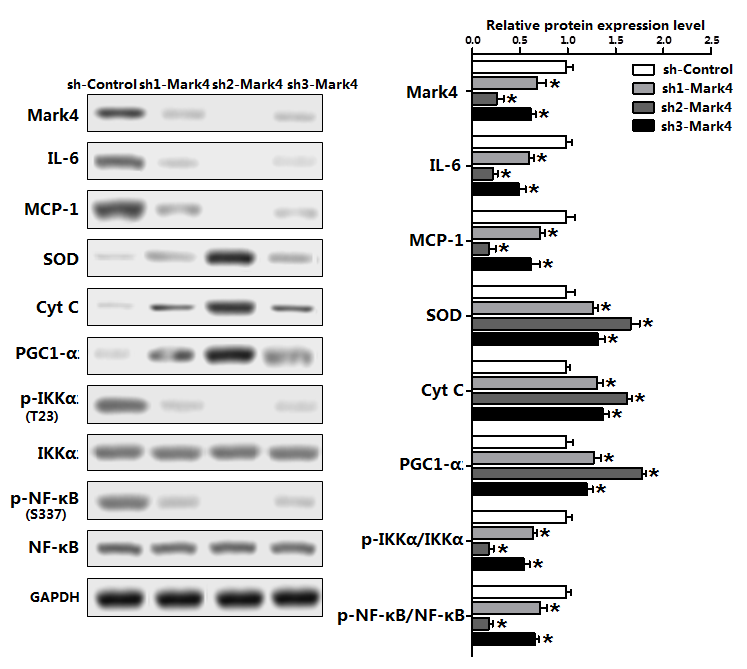
**Fig.S5 Relative protein expression levels in adipocytes transfected with sh-control, sh1-Mark4, sh2-Mark4 and sh3-Mark4 for 48h (Related with Fig.2 and Fig.6).** The protein levels of Mark4, IL-6, MCP-1, SOD, Cyt C, PGC1-α, p-IKKα and p-NF-κB were measured to exclude the shRNA off-target effects (n=3). sh-Control: transfected with negative-shRNA group, sh1-Mark4: transfected with Mark4 interference plasmid group, sh2-Mark4: transfected with Mark4 interference plasmid group targeting different sequence from sh1-Mark4, sh3-Mark4: transfected with Mark4 interference plasmid group targeting different sequence from sh1-Mark4 and sh2-Mark4. Values are means ± SD. vs. control group, * *p* < 0.05.
